# *Pseudomonas aeruginosa* virulence proteins pseudolysin and protease IV impede cutaneous wound healing

**DOI:** 10.1038/s41374-020-00478-1

**Published:** 2020-08-15

**Authors:** Alevoor Srinivas Bharath Prasad, Padival Shruptha, Vijendra Prabhu, Cheruku Srujan, Usha Yogendra Nayak, Calicut Kini Rao Anuradha, Lingadakai Ramachandra, Prasad Keerthana, Manjunath B. Joshi, Thokur Sreepathy Murali, Kapaettu Satyamoorthy

**Affiliations:** 1 Department of Ageing Research, Manipal School of Life Sciences (MSLS), Manipal Academy of Higher Education (MAHE), Manipal, India; 2 Department of Biotechnology, Manipal School of Life Sciences (MSLS), Manipal Academy of Higher Education (MAHE), Manipal, India; 3 Department of Biotechnology, Manipal Institute of Technology, Manipal Academy of Higher Education (MAHE), Manipal, India; 4 Department of Pharmaceutics, Manipal College of Pharmaceutical Sciences, Manipal Academy of Higher Education (MAHE), Manipal, India; 5 Department of Pathology, Kasturba Medical College (KMC), Manipal Academy of Higher Education (MAHE), Manipal, India; 6 Department of Pathology, Yenepoya Medical College, Mangalore, India; 7 Department of Surgery, Kasturba Medical College (KMC), Manipal Academy of Higher Education (MAHE), Manipal, India; 8 Manipal School of Information Sciences, Manipal Academy of Higher Education (MAHE), Manipal, India; 9 Department of Cell & Molecular Biology, Manipal School of Life Sciences (MSLS), Manipal Academy of Higher Education (MAHE), Manipal, India

**Keywords:** Translational research, Preclinical research

## Abstract

The intricate biological process of cutaneous wound healing is achieved through precise and highly programmed events. Dermal fibroblasts and keratinocytes play a significant role in the process of reepithelialization during wound healing. Pathogenic bacteria such as *Pseudomonas aeruginosa* (*P. aeruginosa)* may delay the proliferative phase of wound repair by secreting their proteins leading to delayed or impaired wound healing. We have analyzed three virulent strains of *P. aeruginosa* isolated from the wound environment which also differed in their ability to produce biofilms. Mass spectrometric analysis of differentially expressed secreted proteins by three virulent strains of *P. aeruginosa* revealed peptides from pseudolysin and protease IV expressed from *lasB* and *prpL* genes. Pseudolysin and protease IV recombinant proteins were tested for their ability to modulate wound healing in several cell types of wound microenvironment in in vitro and in vivo models. Both pseudolysin and protease IV inhibited migration and survival of fibroblasts, keratinocytes, and endothelial cells. In three dimensional spheroid endothelial models and matrigel assays these proteins impeded sprouting and tube formation. In a mouse model of excision wound, pseudolysin and protease IV treatment showed reduced collagen content, inhibited neovascularization and epithelialization, and delayed wound contraction. Furthermore, pseudolysin and protease IV treatment resulted in a significant increase in plasma IL-6 levels when compared to vehicle control and control, suggesting the induction of a state of prolonged inflammation. Taken together, our data indicate pseudolysin and protease IV secreted from biofilm producing and antibiotic resistant *P. aeruginosa* in wound microenvironment produce both local and systemic effects that is detrimental to the maintenance of tissue homeostasis. Hence, these proteins may serve as potential therapeutic targets toward better clinical management of wounds.

## Introduction

*Pseudomonas aeruginosa* (*P. aeruginosa*) is a versatile opportunistic pathogen abundantly present in wound infections [1]. The emerging paradigm of *P. aeruginosa* has evoked much interest in clinical practice throughout the world as it contributes significantly to nosocomial infections [2]. *P. aeruginosa* has the ability to form intractable biofilms and produces a myriad of virulence proteins that confer a significant advantage on its role as a wound pathogen [1]. These virulence factors can degrade the extracellular matrix and alter the cell signaling pathways, thereby facilitating the microorganisms to adhere [3], which subsequently leads to tissue damage and blood vessel invasion. Elastase A, elastase B, protease IV, and alkaline proteases are the major virulence factors produced by *P. aeruginosa* [4]. Elastase B, also called as pseudolysin, is encoded by *lasB* gene that targets the collagen type III from the interstitial extracellular matrix, collagen type IV from the basement membrane, degrades immunoglobulin A and G, and inhibits fibroblast growth during the host-pathogen interaction [3, 5, 6]. Conversion of plasminogen to plasmin is also inhibited by pseudolysin resulting in the accumulation of the fibrin clots in the wound area [7]. Protease IV, on the other hand, is also known as lysyl endopeptidase encoded by the *prpL* gene, which is a 26 kDa iron regulated protein belonging to the chymotrypsin family of proteins [8]. Protease IV triggers the host immune defense system by degrading the fibrinogen, plasminogen, immunoglobulin G and inactivating complement components such as C3 and C1 [9]. However, a clear distinction between these two proteins needs to be biochemically and functionally elucidated. Generally, virulence proteins secreted by pathogens may adversely influence several physiological functions in the host system including wound healing [3].

Wound healing is an intricate and dynamic process encompassing four clearly defined stages such as hemostasis, inflammation, repair, and remodeling. These four stages are intimately interconnected through coordinated cell signaling effects of stromal cells to initiate the local release of various cytokines, chemokines, growth factors, and matrix proteins. Disruption of any of these stages may delay the wound-healing process [10] and these are influenced intrinsically by several local and systemic factors which may play a significant role in delaying the wound-healing process [11]. However, extrinsic factors such as infections are of a major concern which can severely impact the orderly orchestrated plasticity of wound healing. The impact of the biological molecules arising from the pathogen on the host is poorly understood. Our efforts in search of host effects of virulence proteins from *P. aeruginosa* in vitro and in vivo models led us to identify pseudolysin and protease IV as key virulence proteins that determine the delayed wound-healing process.

## Materials and methods

### Cell culture

Human foreskin fibroblasts were isolated after obtaining the ethical clearance from the institutional ethics committee, Kasturba Medical College, Manipal Academy of Higher Education, Manipal, India. Written informed consent from the donors were obtained. Isolation of fibroblasts was performed using skin epidermis (human dermal fibroblasts (HDF)), characterized and cells were grown in Dulbecco’s Modified Eagle Medium (DMEM) with 10% fetal bovine serum (FBS) (Himedia, Mumbai, India). Human keratinocytes (HaCaT) cell lines obtained from ATCC and cells were grown in DMEM supplemented with 10% FBS. Human endothelial cells (human umbilical vein endothelial cells—HUVECs) were cultured using endothelial cell growth medium (ECGM) containing growth supplements (Promo cell GMBH, Germany) as described earlier [12].

### Microorganisms, vectors, media

*P. aeruginosa* strains used in the study were isolated from the wounds of diabetic foot ulcer individuals [1]. Samples were collected from consenting individuals after obtaining the Institutional Ethics Committee approval of Kasturba Medical College, MAHE, Manipal. *P. aeruginosa* was grown in tryptic soy broth (TSB) with 1% glucose at 37 °C under aerobic conditions. *E. coli* DH 5α (Novogen, Germany) was used for cloning experiments. Vector pET 28 a (+) (Novogen, Germany) and *E. coli* BL21 (DE3) (Novogen, Germany) strain was used for the protein expression. 2X YT medium containing Kanamycin was used to grow the bacteria with the recombinant construct at 37 °C. The reference strain used in the study was *P. aeruginosa* ATCC 27853.

### Analysis of biofilm formation

Biofilm formation of *P. aeruginosa* isolates collected from 50 human subjects was assessed by tissue culture plate method [13]. Overnight grown cultures of *P. aeruginosa* were diluted in TSB to an optical density of 0.1 at 600 nm. Cultures were then transferred to the individual wells of a sterile polystyrene flat bottom 96-well culture plates. The plates were incubated for 24 h at 37 °C in a static incubator and post incubation period; the wells were washed with phosphate buffered saline (PBS). 0.1% crystal violet was used to stain the biofilms formed and solubilized using 30% glacial acetic acid. The amount of biofilm formed was quantified using plate reader (Varioskan^TM^ Thermo Scientific) by measuring the absorbance at 570 nm [1].

### Confocal laser scanning microscopy (CLSM)

Biofilms formed by *P. aeruginosa* strains were visualized by confocal microscopy as described earlier [14]. Briefly, biofilms were allowed to form on coverslips, which were placed on a 6-well plate under culture conditions as described above. Coverslips with biofilms were fixed using 4% paraformaldehyde, stained using *Bac*Light bacterial live/dead (SYT09-PI) staining (Thermo Fisher Scientific^TM^, USA) and mounted on a glass slide. Using the excitation/emission maxima of 480/500 nm for SYTO9 and 490/635 nm for PI, mounted slides were observed using confocal microscope (CLSM, Leica, Germany).

### Antibiotic sensitivity test

The antibiotic susceptibility of *P. aeruginosa* isolates was assessed by the Kirby–Bauer disc diffusion method on Muller Hinton agar plate. *P. aeruginosa* strains were inoculated in TSB medium and incubated overnight at 37 °C for 18 h. The cultures were then diluted with TSB to 0.5 McFarland standard and lawn culture of the suspension was made on Muller Hinton agar plates. The discs of sets of ten antibiotics with various concentrations (HiMedia, India) as indicated (Table S1) were placed and incubated for 18–24 h at 37 °C. The interpretation of the results was done as recommended by the Clinical and Laboratory Standards Institute guidelines.

### Preparation of *P. aeruginosa* culture conditioned medium

The biofilm-forming strain of *P. aeruginosa* was grown in TSB with 1% glucose at 37 °C under aerobic conditions for 16–18 h. TSB medium alone was used as blank and incubated as mentioned above. The cultures were then centrifuged and 0.2 µm membrane filter (Millipore, USA) was used to filter the supernatant [15] lyophilized and the concentration of protein was estimated by Lowry method.

### Cytotoxicity assay

HDF and HaCaT cells were seeded at a density of 0.5 × 10^4^ and 1 × 10^4^ cells/mL, respectively, in a 96-well cell tissue culture plates with DMEM (GIBCO-Invitrogen) supplemented with 10% FBS and allowed the cells to attach. The cells were treated with different concentrations of proteins diluted using DMEM medium with FBS for 48 h. MTT (3[-dimethylthiazol-2-yl]-2,5-diphenyl-tetrazolium bromide) was then added and formazan crystals formed were then dissolved and absorbance was measured at 570 nm using a plate reader (Varioskan^TM^ Thermo Scientific, USA).

### Cell migration assay

HDF and HaCaT cells were seeded in 12-well cell culture plates and grown in DMEM containing 10% FBS. Cells were grown to 80% confluency and prior to the experiment, cells were serum starved overnight in the serum-free culture medium. A wound scratch was made on monolayer cells with the help of a sterile 200 μL pipette tip [16]. Cells were then treated as indicated in figure legends and cell migration ability for different cell types was monitored. The area covered after cell migration was analyzed using the ImageJ software (Wayne Rasband, USA).

### Generation of *LasB* and *prpL* plasmid constructs

DNA fragments coding for *lasB* gene, and *prpL* gene (900 bp, 33 kDa, mature pseudolysin and 733 bp, 26 kDa, mature protease IV) were amplified from the genomic DNA of *P. aeruginosa*. The primers used were 5′-ATTGGATCCGAGGCGGGCGGCCCCGGCGG-3′ and 5′-ATACTCGAGGAGCTTACAACGCGCTCG-3′ to amplify *LasB* gene, while primers 5′-TATGGATCCGCCGGCTACCGCGACGG-3′ and 5′-GCCTCGAGGGGCGCGAAGTAGCGGGAGA-3′ were used to amplify *prpL* gene. The purified PCR products and pET 28 a (+) vectors were digested separately using BamHI and XhoI restriction endonucleases. The digested plasmid and PCR products were ligated with T4 DNA ligase and transformed into DH 5α. Recombinant clones were further verified by Sanger sequencing (Applied Biosystems 3130, USA) and transformed into *E. coli* BL21 (DE3) cells to produce recombinant proteins.

### Expression and purification of recombinant proteins

Cells containing the plasmid constructs were grown in 2X YT medium at 37 °C with kanamycin (150 µg/mL), to an absorbance of 0.6–0.9 at 600 nm. The protein expressions were induced by adding 0.1 mM isopropyl-β-d-thiogalactopyranoside (IPTG). After 4 h, cells were lysed using lysis buffer (500 mM NaCl, 50 mM TRIS, 10 mM Imidazole, 10% Glycerol) and disrupted by high-pressure homogenization using APV 1000 homogenizer (SPX, USA) at 800 bar. After centrifugation, the cell-free supernatants were passed through Ni-NTA column and pseudolysin or protease IV was eluted from the Ni-NTA column using elution buffer with varying concentrations of imidazole. The eluted pseudolysin and protease IV were dialyzed against PBS and concentrated using Amicon Ultra—10 kDa concentrator (Merck-Millipore, Germany) at 4000 g at 4 °C for 20 min. The purity of recombinant pseudolysin and protease IV was assessed using 12% SDS-PAGE.

### Identification of secretory proteins by LC-MS

Lyophilized condition medium of *P. aeruginosa* was reconstituted, separated on 12% polyacrylamide gel electrophoresis (SDS-PAGE) and stained with Coomassie blue. The protein bands were excised from the gel and processed as described earlier [17]. Furthermore, tryptic products of proteins were subjected to in Mass spectrometer (6520 accurate-mass Q-TOF, Agilent Technologies, USA) coupled to liquid chromatography. Peptides were resolved in a reverse phase C18 column with 5 µm diameter and 4.6 × 150 mm length (Agilent Technologies, Santa Clara) and eluted in a gradient of 3–70% acetonitrile with a flow rate of 0.4 µL for 45 min. MS/MS spectra were processed using Qualitative Mass Hunter (Agilent Technologies, USA), exported as MASCOT (.mgf), and searched against the Swiss-Prot database using Mascot version 2.3 (Matrix Science Limited, UK).

### Analysis of angiogenic activities in HUVEC cells

#### Tube formation assay

Tube forming assay was performed using HUVECs as described earlier [12]. 60 µl of matrigel was coated on 96-well plate and incubated for 1 h at 37 °C. Cells at a concentration of 2 × 10^4^ cells/well were seeded on the matrigel and treated with pseudolysin and protease IV at different concentrations as indicated and bFGF (10 ng/mL) as positive control. Tube formation was assessed after 6 h of incubation at 37 °C. Images were captured using a microscope (Zeiss Axiovision, Germany) and quantified using ImageJ software.

#### Sprout formation assay

Three dimensional cultures of HUVECs were prepared in 20% methyl cellulose containing ECGM. Cells were suspended as hanging drops on Petri plates and were incubated at 37 °C. The spheroids formed were placed on the collagen matrix and treated with pseudolysin and protease IV with different concentrations as indicated in the figure legend and bFGF (10 ng/mL) as positive control. These spheroids were incubated for further 24 h. The number and the length of the sprouts formed were captured using microscope (Zeiss Axiovision, Germany) and were measured using ImageJ software.

#### In vivo wound induction in mice

In all, 6–8-week-old Swiss albino healthy male mice (25–30 g) were obtained from an inbred colony maintained at the central animal facility at MAHE under the controlled conditions of temperature (23 ± 2 °C), humidity (50 ± 5%), and light cycle (14 h of light and 10 h dark). Study was approved by the Institutional Animal Ethics Committee (IAEC/KMC/101/2017). The dorsal part of the animal skin was shaved and anesthetized by intraperitoneal injection with the cocktail of 65 mg/kg Ketamine (Aneket, Neon Laboratories, India) and 8 mg/kg Diazepam (Calmpose, Ranbaxy Laboratories, India). Excisional wounds (1.5 cm^2^) were created on animals as described earlier [18]. Each animal was maintained in a separate polypropylene cage.

#### Gel formulation and topical application of proteins

One percent Carbopol 934 (500 kDa), polymer gel was used as the vehicle control. Triethanolamine was used to neutralize the pH of the gel. Protein loaded 1% carbopol 934 gel formulation was prepared by taking 1 mg/mL of proteins. Post formulation, the stability of the protein was measured at different time points and protein concentrations were estimated by the Lowry method. Homogeneity, texture, and transparency of the formulation were tested after the polymerization [19]. The pH of the gel was measured using a pH meter (Eutech, Instruments, Thermo Scientific, USA).

Mice (*n* = 96) were stratified into four groups; each group containing 24 animals. Group 1: control; Group 2: vehicle control (treated only with 50 µg of carbopol 934); Group 3: pseudolysin treatment (50 µg); and Group 4: protease IV treatment (50 µg). For both vehicle control and treatment groups (Groups 3 and 4), topical application of the formulation was applied twice daily. Animals assigned to the control group were devoid of any treatment throughout the experiment. Mice in each group were further used for performing the following analysis: kinetics of wound healing (*n* = 6/group); histological analysis (*n* = 9/group); biochemical parameters (*n* = 9/group).

#### Assessment of wound healing and contraction

Wound healing was monitored in 24 animals (six from each group) by taking the photographic images of the wounds on every alternate day using Nikon Coolpix P7700 camera at a fixed height and measured using ImageJ software until the wound closure. The wound area on day 1 was considered as 100% and the healing time was noted over a period; mean wound healing time for animals for each group was measured, documented, and represented.

#### Histological studies

Histological analysis was performed on the granulation tissue/skin samples taken from three animals of each experimental group on the 6th, 12th, and 18th day post wounding following euthanization (*n* = 9/group). The granulation tissues were initially fixed in 10% formalin. Tissues were then processed and embedded in paraffin wax for further histological evaluation. From each tissue block, 5-µm thickness tissue sections were taken using a microtome (Leica RM2125RT, Germany), stained with haematoxylin–eosin (H&E) and Masson trichrome (MT). Qualitative assessment of inflammatory state, epithelialization, neovascularization, and collagen synthesis was performed by a pathologist in a blinded fashion. Quantitative assessment of collagen deposition was performed on MT stained tissue sections by digitally analyzing the images using the TissueQuant software (https://manipal.edu/sois/research/TQ_Tool.html).

#### Immunohistochemistry

Paraffin-embedded sections (5 µm) were deparaffinized and rehydrated. Sections were then subjected to antigen retrieval and stained according to the manufacturer’s manual (Cell Signaling Technologies, USA). Assessment of cell proliferation was done by staining tissue sections with rabbit anti-Ki67 (1:400) and the angiogenic process was assessed by staining the tissue sections with rabbit anti-CD31 primary antibodies (1:200 diluted) (Cell Signaling Technologies, USA) separately for overnight at 4 °C. These tissue sections were then stained with biotinylated secondary antibodies at room temperature for 1 h and incubated with avidin-HRP solution. The tissue sections were then developed with 3, 3′-diaminobenzidine solution and counterstained with haematoxylin. Tissue sections were then observed and scored microscopically (Olympus BX51, Japan). The proliferation index and quantification of neo vessel formation were calculated on Ki67 and CD31 stained cells by digitally analyzing the images using the TissueQuant software.

#### Quantification of IL-6, lipid peroxidation, and collagen

Blood and granulation tissue samples from three animals of each experimental group were collected on 6th, 12th, and 18th day post wounding following euthanization (*n* = 9/group). Blood samples were then centrifuged at 2000 g for 10 min and plasma samples were stored in −80 °C for further analysis. IL-6 levels were estimated using ELISA Kit (R&D systems, USA) according to the manufacturer’s manual. Granulation tissues were snap-frozen and stored at −80 °C for the hydroxyproline and lipid peroxidation estimation. For lipid peroxidation assay, tissue samples were homogenized using homogenization buffer (0.1% SDS, 1% Tween-20, 5 mM EDTA, 1X PBS) containing protease inhibitor (Roche Diagnostics, Switzerland). Tissue homogenate was also prepared in 1 mM ascorbic acid and lipid peroxidation in tissue homogenates was measured as thiobarbituric acid reactive substances (TBARS) using UV–visible spectrophotometer, the wavelength of 532 nm. Estimation of hydroxyproline was performed from the tissue homogenate as described earlier [20]. The absorbance of the sample was measured using UV–visible spectrophotometer at 550 nm (Varioskan™, Thermo Scientific, USA).

#### Statistical analysis

The data were presented as mean ± SD or otherwise indicated. Statistical analysis was done using one-way analysis of variance that was used for Bonferroni’s multiple comparisons test. Statistical significances were considered at *p* value: *p < 0.05, **p < 0.01, and ***p < 0.001. Statistical analysis was performed using GraphPad Prism 8.0 (San Diego, USA).

## Results

### *Pseudomonas* strains from wound microenvironment differ in their ability to produce biofilms and resistance against antibiotics

**Fig. 1:**
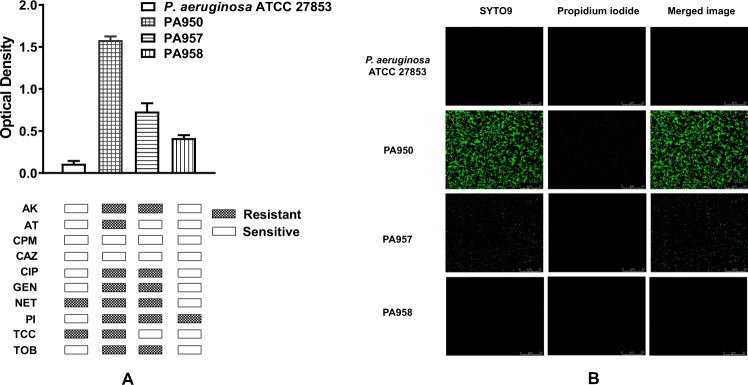
*P. aeruginosa* strains show varied ability to produce biofilms and resistance against antibiotics. Biofilm formation and antibiotic resistance pattern of *P. aeruginosa* ATCC 27853 strain, high, moderate, and low biofilm-forming strains of *P. aeruginosa*. **a** Biofilm production of *P. aeruginosa* strains and antibiotic resistance pattern of the corresponding strains. **b** Confocal microscope images of biofilm formation by ATCC 27853 strain, high, moderate, and low biofilm-forming strains of *P. aeruginosa* after 24 h (×100 magnification). Scale bars correspond to 25 µm.

*Pseudomonas* strains isolated from 50 consecutive individuals diagnosed with diabetic foot ulcer were initially stratified based on their ability to produce biofilms. Biofilm-forming strains were identified based on the absorbance value after crystal violet staining and confocal microscopy. The strains were categorized as high (OD > 0.24), moderate (OD 0.12–0.24), and low (OD < 0.12) biofilm producers. Among 50 strains of *P. aeruginosa* tested, 15 were considered as low, 9 as moderate, and 26 as high biofilm-forming organisms, respectively (Fig. S1). Based on the antimicrobial resistance profile (Fig. S2) for 10 different antibiotics, the 50 strains were classified into three groups as Group 1 strains which were resistant to 3 or lesser antibiotics, Group 2 strains were resistant to 4–7 antibiotics, and Group 3 strains which showed resistant to more than 7 antibiotics. We found that 27 of the strains belonged to Group 1, 10 strains to Group 2, and 13 strains to Group 3. For further analysis, one strain from each of the above three groups was chosen. Strain PA950 was chosen from Group 3 as it showed the highest biofilm-forming ability, PA957 was chosen from Group 2 with moderate biofilm-forming ability, while PA958 from Group 1 showed lesser biofilm-forming ability (Fig. 1a).

### Analysis of biofilm formation by confocal laser microscopy

Representative images of biofilm formed by various strains of *P. aeruginosa* are shown in Fig. 1b. *Bac*Light bacterial live/dead (SYT09-PI) staining was used to describe the architecture of biofilm. Thick and dense layers of biofilm were observed in high biofilm-forming *P. aeruginosa strain*, whereas a thin and sparse layer of biofilm was observed in medium and low biofilm-forming strains of *P. aeruginosa*.

### *Pseudomonas* secretome induced cytotoxic effects and inhibited migration of HDF and HaCaT cells

**Fig. 2:**
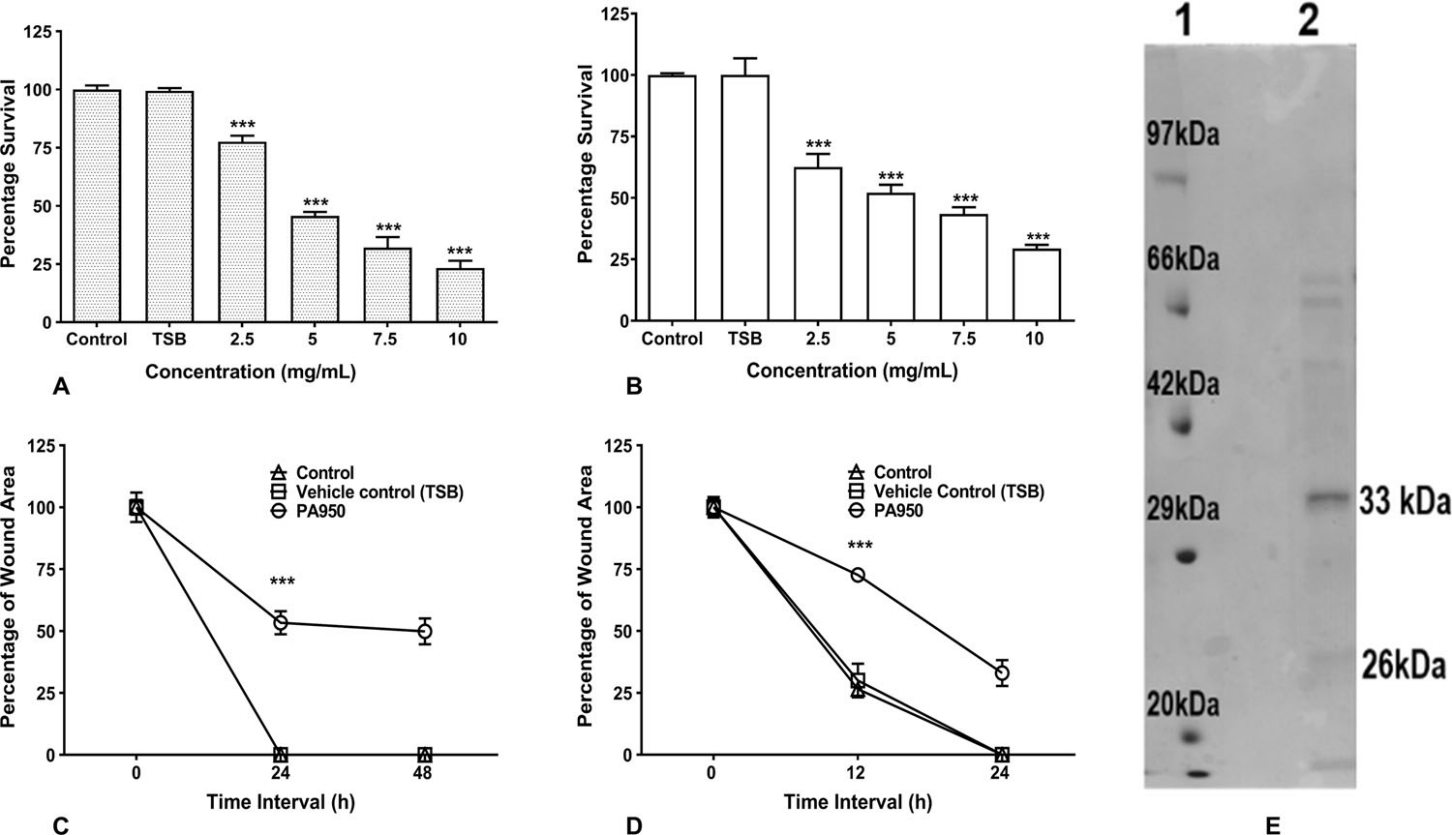
*Pseudomonas* secretome induced cytotoxic effects and inhibited the migration of HDF and HaCaT cells. **a**, **b** Effects of secretory proteins from *Pseudomonas* culture conditioned medium on cell survival of HDF cells and HaCaT cells with concentration ranging from 2.5 to 10 mg/mL by MTT assay after 48 h. **c**, **d** Effect of secretory proteins from *Pseudomonas* culture conditioned medium on cell migration at concentration 2.0 and 1.5 mg/mL on HDF cells and HaCaT cells at different time intervals. Percentage of cell survival and cell migration was calculated and data are shown as mean ± SD, ***p < 0.001 compared to control. **e** Profiling of secretory proteins from *Pseudomonas* culture conditioned medium by 12% SDS-PAGE. Lane 1: protein molecular weight marker (Medium range, GeNei, India), Lane 2: secretory proteins from *Pseudomonas* culture conditioned medium.

Different concentrations of lyophilized *P. aeruginosa* culture condition medium ranging from 2.5 to 10 mg/mL were tested on HDF and HaCaT cells for their cytotoxic effects by MTT assay. The crude secretory proteins from culture conditioned medium of high biofilm-forming strain showed cytotoxicity with IC_50_ value of 3.89 and 5.81 mg/mL on HDF and HaCaT cells, respectively (Fig. 2a, b).

HDF (2.0 mg/mL) and HaCaT cells (1.5 mg/mL) were treated with crude secreted proteins from culture conditioned medium of high biofilm-forming strain to test cell migration in a scratch assay. Kinetic analysis in treated cells showed a significant decrease in wound closure when compared to untreated and vehicle control (TSB) (Fig. 2c, d). Besides, high biofilm-forming strain showed a significant reduction in cell migration in both HDF and HaCaT cells. Protein-treated HDF and HaCaT cells showed relatively slower migration of 50% and 67%, respectively, whereas vehicle controls showed 100% cell migration at the end of 48 and 24 h similar to controls. These results suggest that *Pseudomonas* secretome may possess cytotoxic and anti-migratory properties.

### Profiling of secretory proteins in *P*. *aeruginosa* culture conditioned medium

The lyophilized *P. aeruginosa* culture conditioned medium of high biofilm-forming strain was subjected to SDS-PAGE to visualize secretory proteins. Secretory proteins of high biofilm-forming strain with significant cytotoxicity and reduced cell migration potential showed prominent band with the molecular mass of 33 kDa and less intensified band with the molecular mass of 26 kDa (Fig. 2e). These bands were isolated and subjected to peptide mass finger printing LC/MS/MS analysis and tryptic digested peptides, which led to the identification of pseudolysin and protease IV. Peptide matched with pseudolysin and protease IV with a significant score of 2157 and 975, respectively (Table 1).

**Table 1:** Mass spectroscopic profiles of pseudolysin and protease IV proteins from *Pseudomonas* culture conditioned medium (peptides that matched are in bold).

Sample	Mascot score	Protein name	Peptide sequence	MW
*P. aeruginosa* culture conditioned medium	2157	Pseudolysin	GGPGGNQKIG K**YTYGSDYGP LIVNDR**CEMD DGNVITVDMN SSTDDSKTTP FR**FACPTNTY KQVNGAYSPL NDAHFFGGVV FK**LYR**DWFGT SPLTHK**LYMK VHYGRSVENA YWDGTAMLFG DGATMFYPLV SLDVAAHEVS HGFTEQNSGL IYR**GQSGGMN EAFSDMAGEA AEFYMRGKND FLIGYDIKKG SGALRYMDQP** **SR**DGR**SIDNA SQYYNGIDVH HSSGVYNRAF YLLANSPGWD TRKAFEVFVD** **ANR**YYWTATS NYNSGACGVI RSAONR**NYSA ADVTR**AFSTV GVTCPSAL	53882
*P. aeruginosa* culture conditioned medium	975	Lysyl endopeptidase (protease IV)	QVSTFADSLY KAGYRDGFGA SGSCEVDAVC ATQSGTR**AYD NATAAVAK**MV FTSSADGGSY ICTGTLLNNG NSPKRQLFWS AAHCIEDQAT AATLQTIWFY NTTQCYGDAS TINQSVTVLT GGANILHRDA K**RDTLLLELK** RTPPAGVFYQ GWSATPIANG SLGHDIHHPR GDAK**KYSQGN VSAVGVTYDG HTALTRVDWP** **SAVVEGGSSG SGLLTVAGDG SYQLRGGLYG GPSYCGAPTS** **QRNDYFSDFS** **GVYSQISR**YF AP	48582

### Ammonium sulfate precipitated and purified fractions containing pseudolysin and protease IV modulate cytotoxicity and migration in cell types

Extraction of pseudolysin and protease IV from *P. aeruginosa* culture conditioned medium was performed by ammonium sulfate precipitation method. The extracted secretory proteins were separated on a 12% SDS-PAGE (Fig. S3a). Upon ammonium sulfate precipitation, two major proteins corresponding to 33 and 26 kDa were observed in abundance along with proteins with <20 kDa in minor amounts. Mass spectrometry analysis of these abundant proteins revealed the peptides to be pseudolysin and protease IV with the best score of 3350 and 1061, respectively (Table S2). Due to close molecular weights between these two proteins, the individual isolation or separation of proteins was not achieved even after size-exclusion chromatography. Further to assess the cytotoxic effects of these secretory proteins on HDF and HaCaT cells, we performed concentration dependent MTT assay. On HDF, cytotoxic effects were observed with an IC_50_ value of 22.21 µg/mL (Fig. S3b), while IC_50_ value was found to be 11.23 µg/mL (Fig. S3c) in HaCaT cells. Under normal culture conditions, HDF cells required 48 h for wound closure and treating cells with these fractions with concentrations of 5.0 and 7.5 µg/mL significantly hindered migration to 70% and 48%, respectively (Fig. S3d). Anti-migratory properties of these protein fractions were also observed in HaCaT cells where only 45% of wound closure at the end of 24 h post wounding (5.0 and 7.5 µg/mL), whereas control showed complete wound closure at the end of 24 h (Fig. S3e).

### Expression of recombinant virulence proteins

**Fig. 3:**
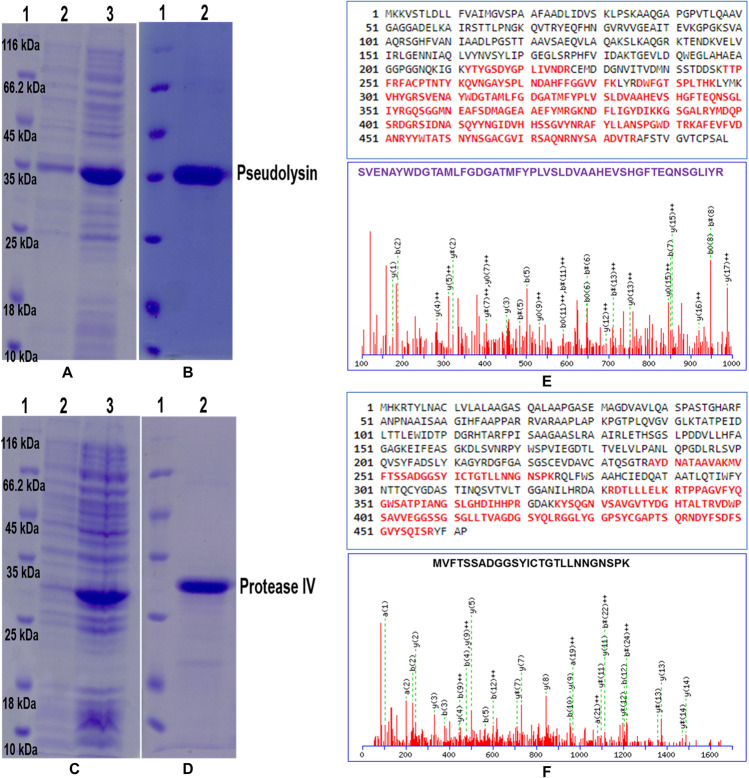
Recombinant mature pseudolysin and protease IV separated on 12% SDS-PAGE and representative sequence of peptides identified by MS/MS analysis. **a** Isolation of recombinant 33 kDa mature pseudolysin (uninduced and induced) expressed in *E. coli* BL21 (DE3) cells. Lane 1: protein molecular weight marker (Thermo ScientificTM 26610, USA), Lane 2: uninduced protein sample, Lane 3: induced protein sample. **b** Purified recombinant mature 33 kDa pseudolysin on 12% SDS-PAGE. Lane 1: protein molecular weight marker, Lane 2: 33 kDa mature pseudolysin. **c** Isolation of recombinant 26 kDa mature protease IV (uninduced and induced) expressed in *E. coli* BL21 (DE3) cells. Lane 1: protein molecular weight marker, Lane 2: uninduced protein sample, Lane 3: induced protein sample. **d** Purified recombinant 26 kDa mature protease IV on 12% SDS-PAGE. Lane 1: protein molecular weight marker, Lane 2: 26 kDa mature protease IV. **e**, **f** Peptide sequence obtained by MS/MS analysis of recombinant pseudolysin and protease IV, respectively.

The *lasB* gene encoding for the mature active pseudolysin and *prpL* gene coding for the mature active protease IV were cloned separately into the pET 28 a (+) vector under the control of T7 promoter. Recombinant virulence proteins were efficiently expressed as soluble fraction in *E. coli* BL21 (DE3) cells reaching the optimum levels at 4 h after the induction with IPTG (Fig. 3a, c). The purified recombinant proteins were separated on a 12% SDS-PAGE (Fig. 3b, d) and the bands were identified as pseudolysin and protease IV based on LC/MS/MS analysis (Fig. 3e, f).

### Recombinant pseudolysin and protease IV significantly affected the survival of fibroblasts and HaCaT cells

**Fig. 4:**
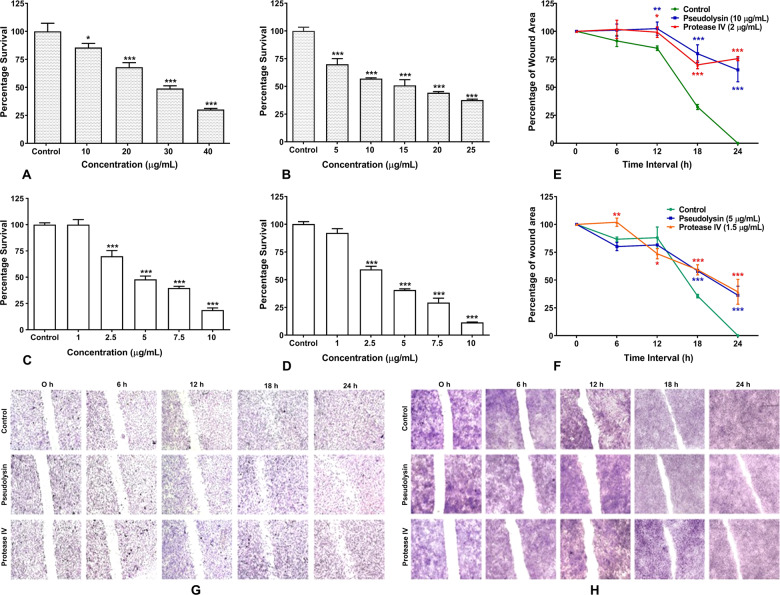
Recombinant pseudolysin and protease IV significantly affected survival and inhibited cell migration. **a–d** Cytotoxic potential of recombinant pseudolysin and protease IV on HDF cells and HaCaT cells. **e**, **f** Effect of recombinant pseudolysin and protease IV on cell migration on HDF cells and HaCaT cells. **g**, **h** Representative images of cell migration of HDF cells and HaCaT cells at various time points. Percentage of cell survival and cell migration was calculated and data are shown as mean ± SD, ***p < 0.001 compared to control.

To evaluate the cytotoxic potential of recombinant pseudolysin and protease IV of *P. aeruginosa* in time-dependent manner, HDF and HaCaT cells were treated with various concentrations. Percentage of cell survival was assessed by MTT assay 48 h post incubation. Recombinant pseudolysin was treated on HDF cells at a concentration ranging from 10 to 40 µg/mL (Fig. 4a) and on HaCaT cells at a concentration ranging from 5.0 to 25 µg/mL (Fig. 4b). HDF cells showed IC_50_ value of 29.29 µg/mL, and it was 13.14 µg/mL for HaCaT cells. Recombinant protease IV was treated on HDF cells at a concentration ranging from 1 to 10 µg/mL (Fig. 4c) and on HaCaT cells at a concentration ranging from 1 to 10 µg/mL (Fig. 4d). IC_50_ values for protease IV for HDF and HaCaT cells were 4.84 and 3.91 µg/mL, respectively. These results suggested that the recombinant pseudolysin and protease IV were capable of inducing the cytotoxicity on the cells involved in wound healing.

### Recombinant pseudolysin and protease IV inhibited cell migration

**Fig. 5:**
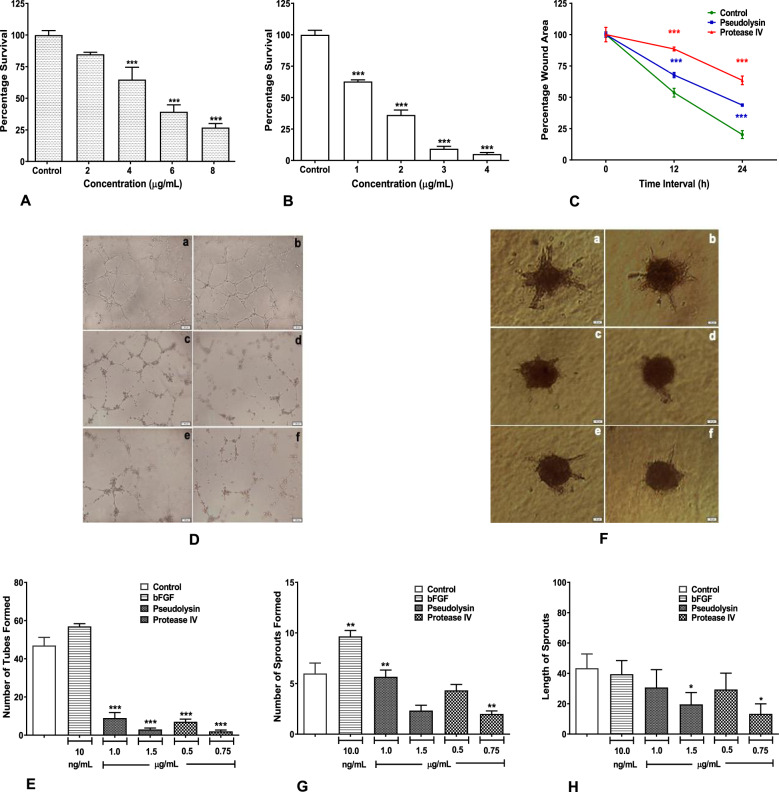
Recombinant pseudolysin and protease IV inhibited angiogenic activities. **a**, **b** Cytotoxic potential of recombinant pseudolysin and protease IV on human endothelial (HUVEC) cells. Percentage of cell survival was calculated and data were shown as mean ± SD, ***p < 0.001 compared to control. **c** Effect of recombinant pseudolysin and protease IV on the migration of HUVECs. The percentage of wound closure was calculated at different time intervals. **d**, **e** Tube formation assay. **f**–**h** Sprouting assay. a Control, b bFGF 10 ng/mL, c pseudolysin 1.0 µg/mL, d pseudolysin 1.5 µg/mL, e protease IV 0.5 µg/mL, f protease IV 0.75 µg/mL. Data were shown as mean ± SD, ***p < 0.001 compared to control.

Recombinant pseudolysin and protease IV showed a significantly inhibited cell migration on HDF and HaCaT cells. Quantitative analysis at different time intervals showed a progressive decrease in the wound closure rate in recombinant pseudolysin and protease IV treated cells when compared to control. The concentration of recombinant pseudolysin used was 10 µg/mL for HDF (Fig. 4e) and 5 µg/mL for HaCaT cells (Fig. 4f). A significant reduction in wound closure rate of 65% on HDF cells and 36% on HaCaT cells was observed after 24 h post wounding compared to controls. HDF and HaCaT cells were also treated with recombinant protease IV at 2 µg/mL (Fig. 4e) and 1.5 µg/mL (Fig. 4f), respectively. Protease IV showed significant reduction in wound closure rate of 75% for HDF cells and 39% for HaCaT cells. Taken together, our results indicated that pseudolysin and protease IV inhibited the migratory property of HDF and HaCaT cells.

### Recombinant pseudolysin and protease IV inhibit angiogenic activities

We investigated the effect of recombinant pseudolysin and protease IV on angiogenesis using HUVEC cells. Initial assessment of cytotoxicity by pseudolysin and protease IV was assessed by performing MTT assay using HUVECs. HUVECs were treated with recombinant pseudolysin and protease IV at a concentration ranging from 2 to 8 µg/mL (Fig. 5a) and 1 to 4 µg/mL, respectively (Fig. 5b), and the IC_50_ values of 5.19 and 1.65 µg/mL, respectively, were obtained. In vitro cell migration analysis of HUVEC cells showed a significant reduction in wound closure in cells treated with pseudolysin at 1 µg/mL and protease IV at 0.5 µg/mL when compared to control at the end of 24 h post wounding (Fig. 5c). Furthermore, HUVECs tube formation (Fig. 5d, e) and sprouting (Fig. 5f–h) were significantly inhibited by both pseudolysin and protease IV. These results demonstrate that the pseudolysin and protease IV have an ability to inhibit angiogenesis in vitro.

### Wound contraction assays indicated a significant delay in the wound-healing process by recombinant pseudolysin and protease IV

After confirming cytotoxic and anti-migratory properties of recombinant pseudolysin and protease IV, we investigated the influence of these proteins on wound contraction in in vivo mouse models. Characterization of carbopol 934 gel impregnated with pseudolysin and protease IV was performed after 2 h of gel preparation. Functional studies confirmed the texture of carbopol 934 gel as homogenous, translucent, and smooth. The pH was observed to be ranging from 7.0 to 7.4. Carbopol 934 powder formed a gel upon mixing with recombinant pseudolysin and protease IV, facilitating convenient for topical administration. The protein content was found to be 95.37 ± 2.12% for pseudolysin gel and 96.52 ± 3.74% for protease IV gel indicating the stability and content uniformity in the gel. The characteristics of carbopol are represented in Table 2.

**Table 2:** Characterization of carbopol gel 934.

Appearance	Transparency	Homogeneity	Texture	pH	Drug content
	Translucent	Highly homogeneous (no crystals observed)	Smooth	7.0–7.4	95.37 ± 2.12% 96.52 ± 3.74%

**Fig. 6:**
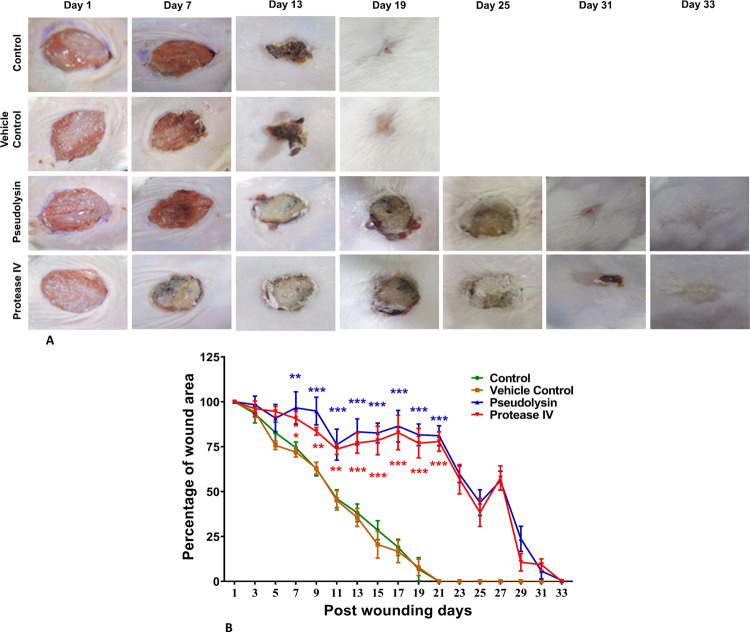
Wound contraction assays indicated significant delay in the wound-healing process by recombinant pseudolysin and protease IV. **a** Representative photographic images of control, vehicle control, pseudolysin, and protease IV treated groups showing a reduction in wound area post wounding. **b** Wound contraction kinetics of different experimental groups expressed as percentage of the wound area. Images were taken every alternative day and analyzed by ImageJ software. Data represented are mean ± SEM. ***p < 0.001 compared to control.

Wound healing progression was monitored by taking digital images on every alternative day until complete wound closure (Fig. 6a). The control and vehicle control showed progressive healing and the complete wound closure was observed on 21st day post wounding. Pseudolysin and protease IV significantly (*p* < 0.001) delayed wound contraction when compared to vehicle control and control groups (Fig. 6b). Pseudolysin treated animals and protease IV treated animals took 31 and 33 days, respectively, for the complete closure of the wound. During the entire study period, wounded animals were healthy and there were no adverse effects.

**Fig. 7:**
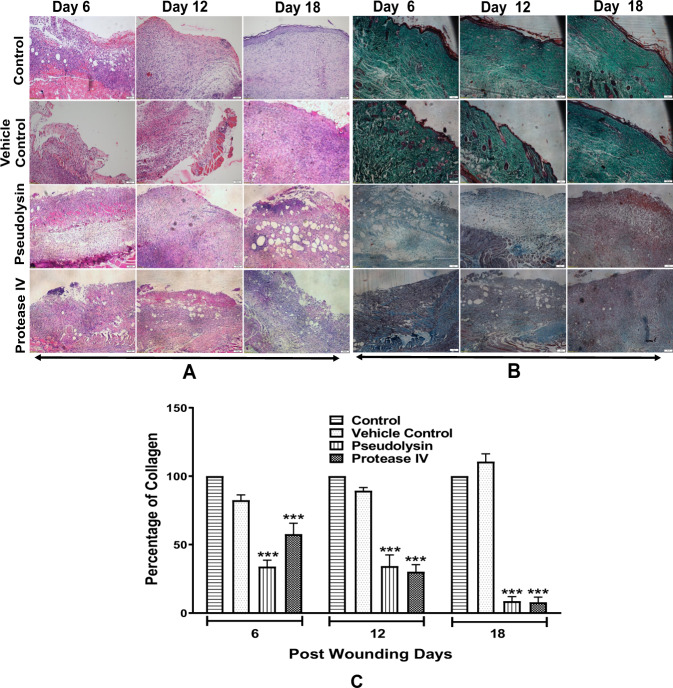
Representative images of histology of granulation tissue of different experimental groups at days 6, 12, and 18 post wounding at ×10 magnification. **a** H&E staining. **b** Masson trichrome staining. Scale bars: 100 μm. **c** Quantitative analysis of Masson trichrome stained tissue sections by digital image analysis using TissueQuant software. Data represented are mean ± SEM. ***p < 0.001 compared to control.

Initial histological analysis of granulation tissues of control and treated groups was performed after H&E staining. Wound healing progression was analyzed at different time intervals of 6th, 12th, and 18th day of post wounding (Fig. 7a). On 6th, 12th, and 18th day of wound healing, both the control and vehicle control showed a similar response to edema and on 18th day it was present only on the wound margin. However, in case of the pseudolysin treated group on 6th and 12th day, the edema was present in more than 50% of the tissues analyzed, but on the 18th day, the edema was present at the wound boundaries, whereas protease IV on 6th, 12th, and 18th day, the edema was present in more than 50% of the tissue observed. Presence of leukocytes in both the control groups was mild, while in the case of pseudolysin and protease IV treated groups a moderate number of cells were observed throughout the indicated time points. This suggested a pro-inflammatory condition in treated groups. The presence of granulation tissues and fibroblasts in the pseudolysin and protease IV treated animals showed less evidence compared to the vehicle control and control. While, in control and vehicle control animals, epithelial thickening and cell migration were observed and these were absent in pseudolysin and protease IV treated animals even after the 18th day post wounding. Histological evaluation for collagen was performed on the granulation tissues by MT staining (Fig. 7b). On the 6th day post wounding, both the control and vehicle control groups showed similar levels of collagen present at the focal boundaries of fibroblasts and also around new capillaries. However, pseudolysin and protease IV treated groups did not show any evidence of collagen synthesis. On the 18th day post wounding, a moderate amount of collagen levels was observed in untreated and vehicle control groups, whereas mild collagen was observed in pseudolysin treated groups and there was no evidence of collagen in protease IV treated groups.

Image analysis of MT stained granulation tissues showed a significant reduction in collagen levels in pseudolysin and protease IV treated groups when compared to vehicle control and control. We observed a 66.2% reduction in collagen levels on day 6, 65.7% reduction on day 12, and 91.3% reduction on day 18 in pseudolysin treated group, while in protease IV treated group the corresponding values were 42.5%, 69.8%, and 92.1% reduction on days 6, 12, and 18, respectively (Fig. 7c).

**Fig. 8:**
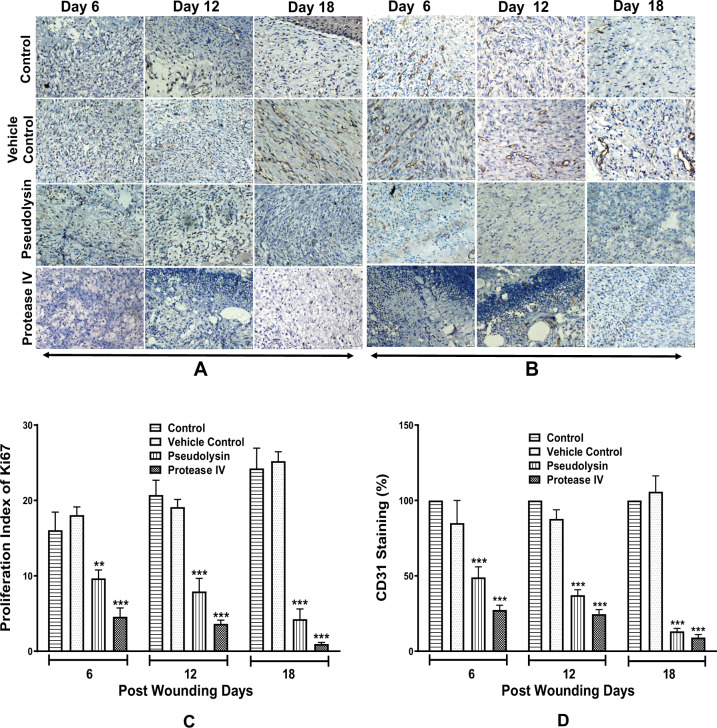
Representative images of immunohistochemical examination of granulation tissue at days 6, 12, and 18 post wounding at ×10 magnification. **a** Granulation tissues were stained with Ki67. **b** Granulation tissues were stained with CD31. Scale bars: 100 μm. **c**, **d** Proliferation index of Ki67 stained tissue sections and quantification of neo vessel formation by digital image analysis using TissueQuant software. Data represented are mean ± SEM. ***p < 0.001 compared to control.

Ki67 and CD31 staining was performed to investigate cell proliferation and neo-angiogenesis (Fig. 8a, b). We observed a significant decrease in the proliferation of fibroblasts and keratinocytes when compared to control groups. Vascular endothelial cell proliferation was detected in granulation tissues of control groups on 6th day post wounding with the subsequent increase in the number of proliferating endothelial cells resulting in vessel rich granulation tissue on 12th and 18th day post wounding, whereas significantly less vascularization was observed throughout the wound-healing process in recombinant protein-treated groups. A significant decrease in micro vessel density was observed in both pseudolysin and protease IV treated animals compared to the untreated and vehicle control groups.

Pseudolysin and protease IV treatment resulted in a significant reduction in proliferation of fibroblasts and keratinocytes in granulation tissues when compared to vehicle control and control groups. Proliferation index was 9.6% on day 6, 7.9% on day 12, and 4.2% on day 18 in pseudolysin treated group, while in protease IV treated group we observed 4.5, 3.6, and 0.9% on days 6, 12, and 18 when compared to control group (Fig. 8c).

Similarly, image analysis was performed on CD31 stained granulation tissues to measure the vessel density (Fig. 8d). We observed a significant reduction in vessel density in granulation tissues treated with pseudolysin and protease IV when compared to vehicle control and control groups. There was 51 and 72.6% reduction in vessel density on day 6 (*p* < 0.001), 62.8 and 75.4% reduction on day 12 (*p* < 0.001), and 86.8 and 90.8% reduction on day 18 (*p* < 0.001) in pseudolysin and protease IV treated groups compared to control.

**Fig. 9:**
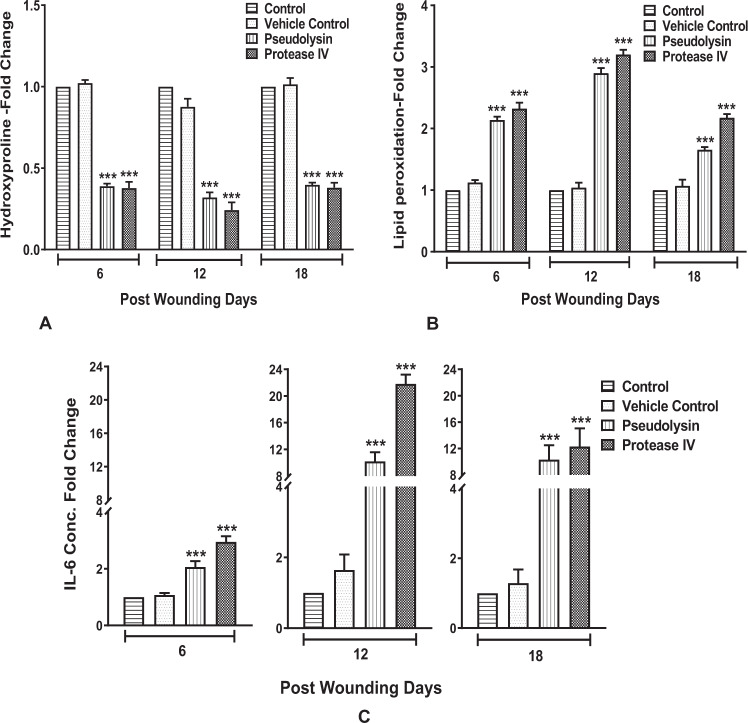
Quantification using biochemical tests. **a** Hydroxyproline content (hydroxyproline/mg of granulation tissue). **b** Lipid peroxidation levels (lipid peroxidation/mg of granulation tissue) of control, vehicle control, pseudolysin, and protease IV treated groups upon wound healing at different time points. **c** IL-6 levels by ELISA. Change in parameters compared to controls was expressed as fold change at different post wounding days. Data represented are mean ± SD, ***p < 0.001 compared to control.

Furthermore, we estimated hydroxyproline levels in granulation tissues to assess collagen content. The results of the hydroxyproline concentration in the granulation tissue displayed a similar pattern to those results observed in the histological analysis with reduced collagen content in protein-treated groups (Fig. 9a). The rise in hydroxyproline content was observed on days 6 and 12 and gradually stabilized on the 18th day in vehicle control and control groups. On the other hand, granulation tissues of the pseudolysin and protease IV treated animals showed significantly lower levels of hydroxyproline at all the time points (*p* < 0.001).

### Pseudolysin and protease IV enhance oxidative stress and pro-inflammatory milieu

Oxidative stress has been reported to modulate wound healing. Hence, we subsequently estimated lipid peroxidation levels as a marker of oxidative stress. On 6th day of post wounding, there was a twofold increase in the TBARS levels in the pseudolysin and protease IV treated tissues compared to vehicle control and untreated groups. On the 12th day of post wounding, there was a 2.5-fold and 2.8-fold increase in the TBARS levels in the pseudolysin and protease IV treated tissues, respectively, compared to both the control groups. On the 18th day of post wounding, there was a 1.8-fold and 2.4-fold increase in the TBARS levels in the pseudolysin and protease IV treated tissues, respectively, compared to both the control groups (Fig. 9b). A significant increase (*p* < 0.001) in TBARS levels was observed in granulation tissues of treated groups on all the post wounding days compared to both control groups.

As we observed the infiltration of leukocytes in animals treated with pseudolysin and protease IV, we tested for IL-6 levels as a sign of inflammation. Pseudolysin and protease IV treated animals showed increased IL-6 levels. The levels of inflammatory cytokine IL-6 were significantly higher in treated groups on 6th, 12th, and 18th day post wounding compared to control (Fig. 9c), on 6th day post wounding, there were 2-fold and 2.9-fold increase in the IL-6 levels in the pseudolysin and protease IV treated tissues, on 12th day there was 10-fold and 21-fold increase, and on 18th day 10-fold and 12-fold increase was observed compared to both the control groups, indicating the prolonged and elevated inflammatory response during the wound-healing process.

Taken together, pseudolysin and protease IV mediated inhibition of wound-healing process was associated with (a) infiltration of leukocytes, (b) reduction in collagen accumulation, (c) decrease in microvessels, (d) increase in oxidative stress, and (e) prolonged and increase in IL-6 levels.

## Discussion

Chronic wounds are a major problem associated with significant morbidity and mortality. The influence of microbial infection on delayed wound healing has remained as one of the key aetiologies and their interactions with host skin microbiota during wound repair remain poorly understood [21]. *P. aeruginosa* forms biofilms and secretes an array of virulence proteins to establish a persistent infection in host cells, which may impact on several processes required in the tissue microenvironment for effective maintenance of homeostasis. Though biofilm-forming strains of *P. aeruginosa* have been shown to be associated with chronic wounds, the exact mechanism remains unclear [22]. The present study was aimed at understanding the role of virulence proteins produced by biofilm producing and multidrug resistant *P. aeruginosa* in cutaneous wound healing. A better understanding of virulence proteins, their biological activities, and underlying mechanisms of their cytotoxicity and its immediate effects on cell migration may provide insight into the wound closure during healing.

In the present study, we investigated two major secretory proteins, pseudolysin and protease IV, from *P. aeruginosa* and established its cytotoxic nature and inhibitory effect on cell migration both in vitro and in vivo. *P. aeruginosa* is one of the highly versatile, opportunistic pathogens, responsible for the nosocomial infections displaying multiple survival strategies such as the development of multidrug resistance, biofilm production, virulence protein production among others [23]. Emerging resistance to multiple classes of antibiotics by the *P. aeruginosa* has been well established over the past few decades and periodical monitoring of susceptibility patterns will provide clinical outcome to various therapeutic approaches. In the present study, 46% of *P. aeruginosa* strains were showing a multidrug resistance pattern (MDR). MDR often attained through several mechanisms, such as acquiring targeted mutations, enhanced multidrug efflux systems, and production of modulatory enzymes [24]. *P. aeruginosa* can exist independently or co-habit along with other microbes to form microcommunities within a secreted matrix of extracellular polymeric substances in the form of biofilms. Biofilms protect bacteria against various types of stresses, including immune responses and antibiotics [25]. Biofilm formation plays an important role in bacterial pathogenesis [26]. *P. aeruginosa* strains tested in our study exhibited a significant correlation between antibiotic resistance and biofilm producing ability which was in agreement with earlier reports [1]. Biofilm formation is the major cause for the persistent infection, they communicate and respond within the biofilm by a process known as quorum sensing. Quorum sensing is a regulator of virulence and biofilm formation in *P. aeruginosa*. *P. aeruginosa* through quorum sensing orchestrate a symphony of virulence factors such as exoproteases, siderophores, exotoxins, and several secondary metabolites, which have been implicated in the pathogenesis [25]. Our results suggested that the bacterial-conditioned medium of high biofilm producing strain of *P. aeruginosa* produced an array of secretory proteins. Furthermore, these secretory proteins showed cytotoxicity and inhibited cell migration on skin cells such as HDF and HaCaT cells in vitro as confirmed by MTT and wound scratch assay. *Pseudomonas* elastase isolated from the culture supernatant was found to be cytotoxic to A549 airway epithelial cells [27]. Collective cell migration is considered as a hall mark of wound repair [28]. In the present study, in vitro scratch assays showed that cells treated with bacterial proteins significantly reduced the rate of wound closure compared to control samples, although kinetics between the cell types varied. Moderate biofilm producing and low biofilm producing strains of *P. aeruginosa* were unable to show any type of such significant observations. This suggested presence or abundance of biomolecules in high biofilm producing strains responsible for virulence properties.

We further identified through mass spectroscopy that the high biofilm-forming strain of *P. aeruginosa* produced pseudolysin and protease IV abundantly in the bacterial-conditioned medium. Earlier studies have also shown the production of secretory proteins elastase B and protease IV in the culture supernatants [9, 29–31]. However, our report is the first study to demonstrate the profiling of secretory proteins in a high biofilm-forming strain from diabetic wound isolate using LC/MS/MS analysis, which resulted in the identification of mature pseudolysin and protease IV proteins that elicited cytotoxicity. Pseudolysin and protease IV have been shown to play a major role in ocular infection, lung infection, disruption of epithelial tight junctions, and degradation of immunoglobulins, complement proteins, and fibrinogen [4].

We next attempted to investigate effects of pure pseudolysin and protease IV in ammonium sulfate fractions on cytotoxicity. Our analysis indicated that these fractions induced cytotoxic effects and inhibited migration even at lower concentrations.

Since both these proteins could not be purified separately, recombinant clones were prepared and cultured to produce individual proteins. This approach offered the possibility to analyze the individual effect of recombinant pseudolysin and protease IV. Several studies reported the synthesis of mature pseudolysin and mature protease IV by recombinant technology using *E. coli* and *Pichia pastoris* with functional characterization of the protease [32, 33]. Furthermore, to confirm the antiproliferative property of *P. aeruginosa* virulence proteins in vitro, recombinant pseudolysin and protease IV were tested for the proliferation potential on HDF, HaCaT cells and HUVECs using MTT assay. A dose-dependent significant decrease in cell viability was observed during the 48-h treatment period. In the present study, a significant decrease in cell migration was observed on HDF, HaCaT cells and HUVECs. Cross talk between fibroblasts and keratinocytes resulting in coordinated proliferation and migration along with the angiogenesis is the vital component in the wound-healing process [34].

Pseudolysin is a 33-kDa extracellular elastase (EC: 3.4.24.26) that belongs to the thermolysin (M4) like family of metallopeptidase and is the most abundant protease secreted by *P. aeruginosa* [32]. Pseudolysin is initially synthesized as 53.6 kDa pre-proenzyme containing a signal peptide, a propeptide, and the C-terminal mature domain. Further cleavage by autoproteolysis in the periplasm results in the mature active protease of 33-kDa [35]. Earlier studies established pseudolysin as a major virulence factor of *P. aeruginosa* playing an important role in its pathogenesis [36]. Pseudolysin has the ability to cause lysis of elastin and some connective tissues and to destroy the blood vessels causing hemorrhage [37, 38].

Protease IV is a 26-kDa Lysine-specific endoprotease (EC 3.4.21.50), initially synthesized as 48-kDa pre-proenzyme containing three domains: signal sequence, propeptide domain, and mature protease. Intracellular cleavage results in the extracellular 26-kDa mature active protease [8]. Earlier studies indicated protease IV as one of the major virulence factors of *P. aeruginosa* for its role in the pathogenicity of keratitis [39]. It is also known to degrade variety of host defense proteins such as fibrinogen, plasminogen, immunoglobulin G and the complement proteins C1q and C3 [9].

Rodents are widely used to understand the dynamics of wound healing due to its low cost and ease of breeding and genetic modifications. Furthermore, the effect of recombinant pseudolysin and protease IV on cutaneous wound healing was evaluated in vivo by making excisional wounds in Swiss albino mice model. We used carbopol hydrogel as vehicle control to facilitate the retention of the proteins in the wound site. Hydrogel-mediated delivery of recombinant pseudolysin and recombinant protease IV represents potentially novel and effective means to study the wound healing progression. In rodents, wound contraction is the significant process in wound healing as their skin is mobile and contains thin sheets of striated muscles between the subcutaneous fat and the dermal layer [40]. Wounds treated with the two recombinant proteins took significantly longer time to heal when compared to that of both vehicle control and control in terms of wound closure and contraction.

The highly dynamic and orchestrated process of wound healing consists of different phases such as hemostasis, inflammation, proliferation, and remodeling [10]. Bacterial virulence factors may significantly delay the process of healing by prolonging inflammatory response, degrading extracellular proteins including collagen, inhibiting the cell proliferation and angiogenesis, and delaying reepithelialization [11]. Histological assessment of the skin is always considered as “gold standard” in diagnosing and demonstrating any degenerative and neoplastic conditions [18]. Ancillary techniques such as special stains (MT staining for collagen) and immunohistochemistry techniques along with the scoring system will help the accurate assessment of wound healing [27]. Histological scoring revealed reduced proliferation of fibroblast, keratinocytes, and endothelial cells resulting in reduced epithelialization in protein-treated groups of animals. Further image analysis of MT stained granulation tissues indicated a significant decrease in the collagen deposition in treatment groups compared to control. Findings of the present study on delayed epithelialization and collagen deposition upon treatment with bacterial secretory proteins were completely corroborated with the earlier published reports in LPS induced delayed wound model [41]. Recent studies demonstrated the action of pseudolysin in destroying human tissues by solubilizing elastin [37]. Taken together, the outcomes of the present investigation clearly establish the role of pseudolysin and protease IV in delaying wound healing by acting on extracellular matrix protein. In the present study, immunohistochemistry staining results revealed decreased proliferation of cells in treatment groups compared to vehicle control and control. A similar study that was performed earlier reported reduction in Ki67 expression indicating lower cellular proliferation in LPS induced delayed wound model [41]. Earlier results observed abnormal keratinocyte and fibroblast migration, proliferation and decreased vascularization in diabetic conditions leading to impaired wound healing [42]. Neovascularization or angiogenesis, the formation of new blood vessels from preexisting capillaries and their penetration toward wound site is an essential component of wound-healing process [43]. In the present study, using HUVEC cells significant cytotoxicity was elucidated and tube formation assay revealed the reduction in angiogenesis which was corroborated by immunohistochemistry staining results indicating the impaired angiogenesis. Collagen is abundantly present in the ECM and is involved in cell adhesion, cell migration, tissue repair, giving tensile strength to the tissue and plays a significant role in the maintenance of tissue homeostasis during the proliferation and remodeling phase of wound healing [44]. Hydroxyproline is an amino acid that plays a significant role in stabilizing the structural conformation of collagen, the principal protein in the ECM of skin. Since hydroxyproline is measured as a surrogate for total collagen and decreased concentration of hydroxyproline in granulation tissue indicates reduced amount of collagen which, in turn, may impair the wound-healing process [45]. Hydroxyproline content in the granulation tissue of pseudolysin and protease IV treated animals showed several fold decrease on days 6, 12, and 18 when compared to control samples.

Malondialdehyde has been widely used and considered as a biomarker for lipid peroxidation [46]. In this study, the tissue homogenates from the wound demonstrated the increase in the levels of MDA indicating increased levels of oxidative stress. Oxidative damage can reduce the cell survival rate, proliferation, differentiation, metabolism and induce apoptosis [47].

**Fig. 10:**
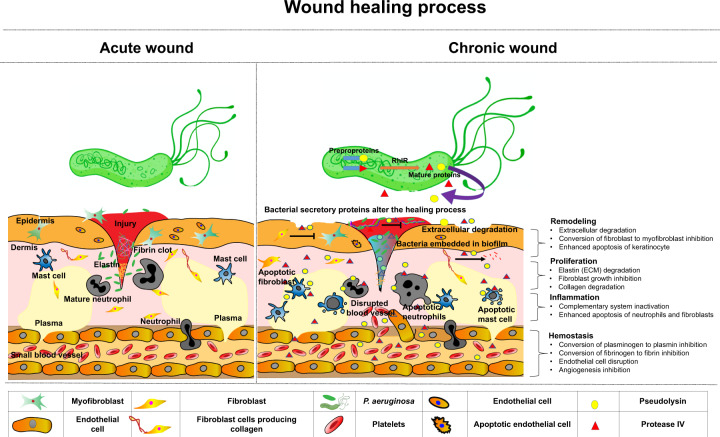
Pseudolysin and protease IV of *P. aeruginosa* delayed cutaneous wound healing through multiple mechanisms. Normal wound healing involves a well-coordinated process involving multiple cell populations, the action of mediators like cytokines and growth factors, and also the extracellular matrix. The mature virulent proteins (pseudolysin and protease IV) produced by multidrug resistant and biofilm-forming *P. aeruginosa* cause apoptosis of neutrophils, macrophages, and mast cells leading to impaired phagocytosis. Prolonged inflammatory phase due to infection and elevated levels of pro-inflammatory cytokines inhibit the proliferation phase and remodeling phase of wound healing. Pseudolysin and protease IV enhance degradation of ECM, inhibit the proliferation of fibroblast, keratinocytes, and endothelial cells. They also cause inactivation of complement proteins and inhibit the conversion of plasminogen to plasmin and fibrinogen to fibrin leading to chronic wound healing.

Collagen is formed in the skin by fibroblasts. High glucose condition induces oxidative stress and may lead to fibroblast dysfunction in diabetic patients and result in decreased collagen production [48]. The present study also confirmed the elevated levels of plasma IL-6 during the wound-healing process indicating the prolonged inflammation process. Cytokines such as IL-6 orchestrate the inflammatory process. Prolonged inflammation may play a critical role in delayed tissue regeneration. Based on the findings of the present study, we propose the action of virulence proteins, pseudolysin, and protease IV in delaying cutaneous wound healing through multiple mechanisms (Fig. 10).

In this study, we have identified two secretory proteins, pseudolysin and protease IV, that can significantly impact wound healing in both in vitro and in vivo models. These proteins induced cytotoxicity and reduced cell migration of cellular components of the wound. Furthermore, we observed both proteins reduced collagen accumulation, increased oxidative damage locally, and enhanced IL-6 levels systemically. We observed pseudolysin and protease IV significantly delayed wound healing and these proteins might serve as therapeutic targets for clinical management of wounds.

## Supplementary information

Supplementary Information
