# Supplementary material for: Pseudomonas aeruginosa virulence proteins pseudolysin and protease IV impede cutaneous wound healing
Source: Lab Invest. 2020 Aug 15;100(12):1532–50. doi: 10.1038/s41374-020-00478-1 (PMC7683349; doi:10.1038/s41374-020-00478-1)
Supplement: Supplementary file 1 — Supplementary Information [file 41374_2020_478_MOESM1_ESM.pdf]

Supplementary Figure:

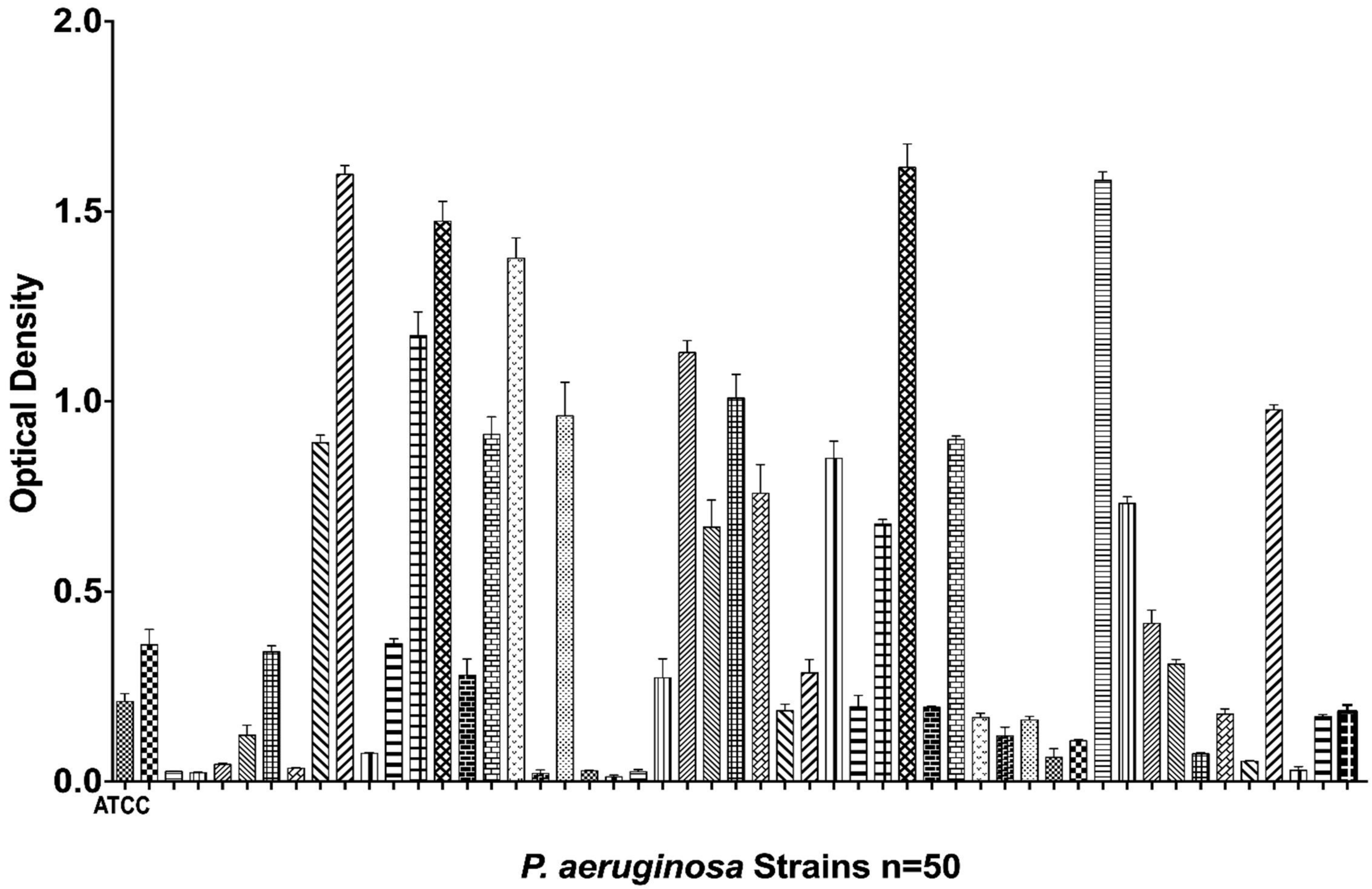

Figure S1: Biofilm forming efficiency of *P. aeruginosa* strains from the diabetic wound subjects. Biofilm formation by individual clinical isolates of *P. aeruginosa* (n=50) and biofilm formation by *P. aeruginosa* ATCC 27853 strain. Data represented are mean ± SD.

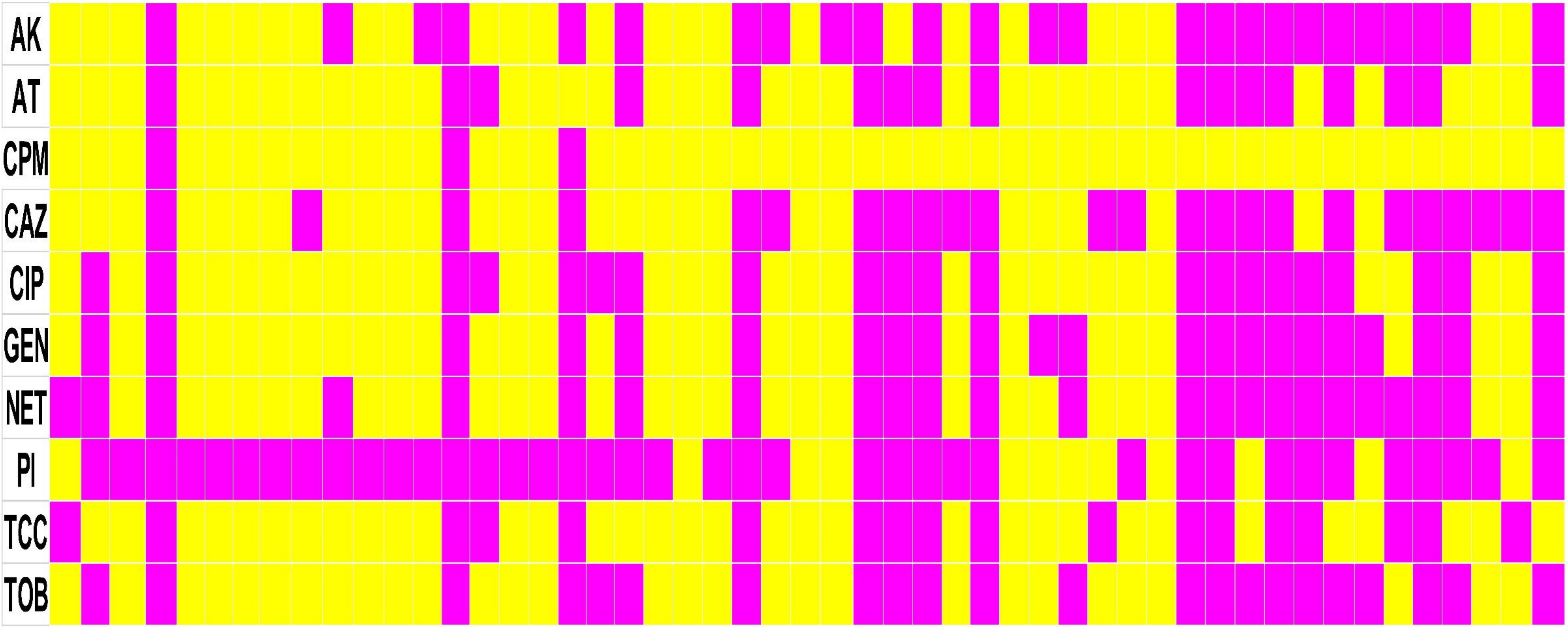

Figure S2: Antibiotic resistance pattern of *P. aeruginosa* strains from the diabetic wound subjects for various antibiotics. Pink and yellow color labels indicating resistant and sensitive strains of P. aeruginosa (n=50) and antibiotic sensitivity of *P. aeruginosa* ATCC 27853 strain.

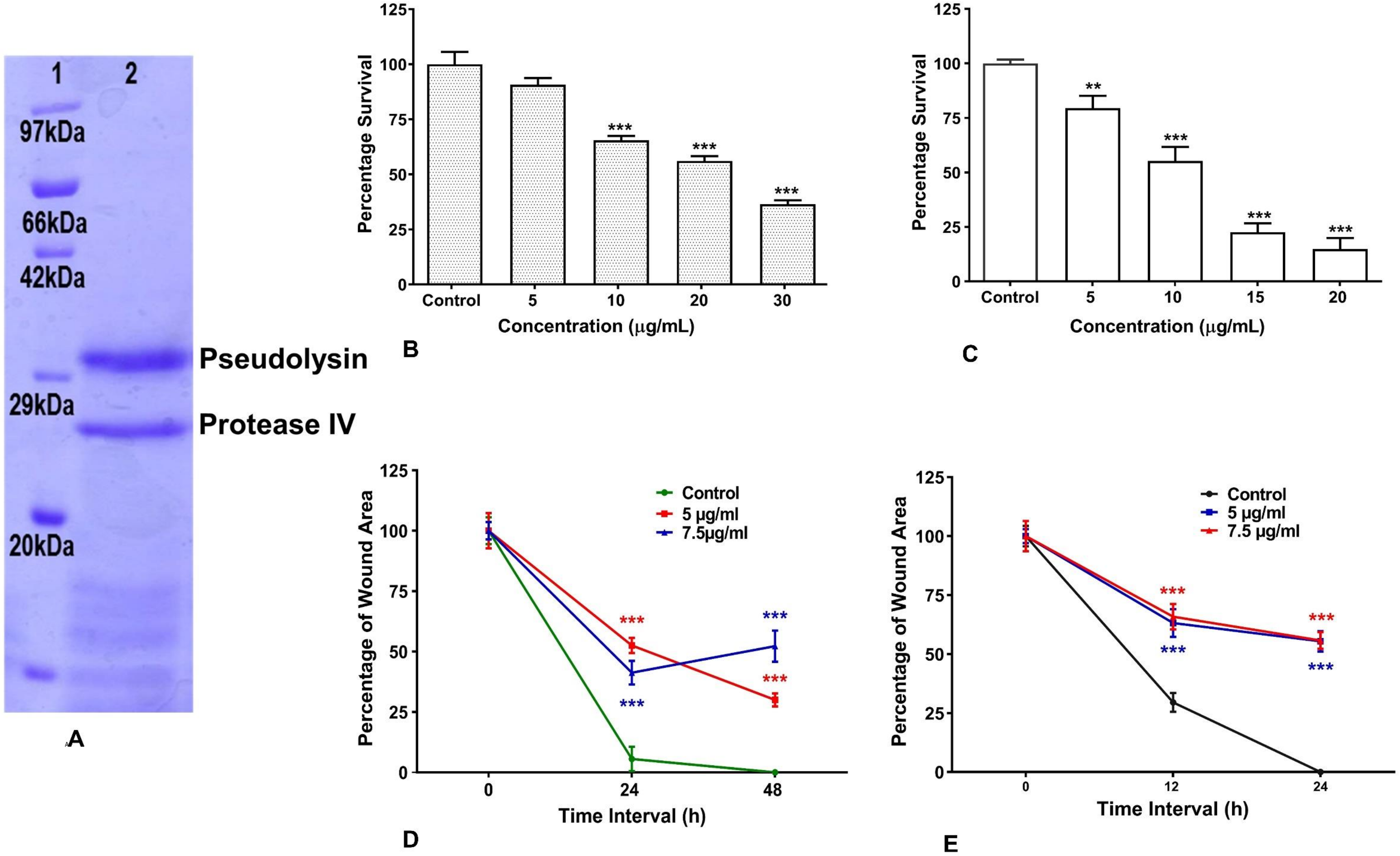

Figure S3. Ammonium sulfate precipitated and purified fractions containing pseudolysin and protease IV modulate cytotoxicity in cell types. **A**. 12 % SDS-PAGE showing secretory proteins in ammonium sulfate precipitated and purified fractions. Lane 1. Protein molecular weight marker (GeNei, India), lane 2. Ammonium sulfate precipitated and purified fraction. **B. & C**. Effect of ammonium sulfate precipitated and purified pseudolysin and protease IV on cell survival of HDF and HaCaT cells with concentration ranging from 5µg/mL to 30µg/mL Cytotoxicity was assessed by MTT assay after 48 h of treatment. **D. & E**. Effect of ammonium sulfate precipitated and purified pseudolysin and protease IV on cell migration of HDF and HaCaT cells. Percentage of cell survival and cell migration was calculated and data are shown as mean ± SD, *** $p < 0.001$ compared to control.

Table S1. **Antibiotic discs and concentrations.**

S.No	Antibiotic	Abreviation
1	Amikacin (30 mg)	AK
2	Azetreonam (30 mg)	AT
3	Cefipime (30 mg)	CPM
4	Ceftazidime (30 mg)	CAZ
5	Ciproflaxacin (5 mg),	CIP
6	Gentamycin (10 mg)	GEN
7	Netilimicin (30 mg)	NET
8	Piperacilin (100 mg)	PI
9	Tetracyclin - Clavlanic Acid (75 mg+10mg)	TCC
10	Tobramycin (10 mg)	TOB

Table S2. Mass Spectroscopic profiles of ammonium sulfate precipitated and purified pseudolysin and protease IV from Pseudomonas culture condition medium (peptides that matched are highlighted).

Sample	Mascot Score	Protein Name	Observed Peptide Sequence	MW
NH_4SO_4 Precipitated & Purified Secretory Proteins	3350	Pseudolysin	GGPGGNQKIG K**YTYGSDYGP LIVNDR**CEMD DGNVITVDMN SSTDDSK**TTP**<br>**FRFACPTNTY KQVNGAYSPL NDAHFFGGVV FKLYRDWFGT SPLTHK**LYMK<br>**VHYGR**SVENA YWDGTAMLFG DGATMFYPLV SLDVAAHEVS HGFTEQNSGL<br>IYR**GQSGGMN EAFSDMAGEA AEFYMRGKND FLIGYDIKKG SGALRYMDQP**<br>**SRDGRSIDNA SQYYNGIDVH HSSGVYNRAF YLLANSPGWD TRKAFEVFVD**<br>**ANRYYWTATS NYNSGACGVI RSAQNRNYSA ADVTRAFSTV GVTCPSAL**	53882
NH_4SO_4 Precipitated & Purified Secretory Proteins	1061	Lysyl Endopeptidase	QVSYFADSLY KAGYRDGFGA SGSCEVDAVC ATQSGTR**AYD NATAAVAK**MV<br>FTSSADGGSY ICTGTLLNNG NSPKRQLFWS AAHCIEDQAT AATLQTIWFY<br>NTTQCYGDAS TINQSVTVLT GGANILHRDA KR**DTLLLELK RTPPAGVFYQ**<br>**GWSATPIANG SLGHDIHHPR** GDAK**KYSQGN VSAVGVTYDG HTALTRVDWP**<br>**SAVVEGGSSG SGLLTVAGDG SYQLRGGLYG GPSYCGAPTS QRNDYFSDFS**<br>**GVYSQISR**YF AP	48582
